# Note on the History of Syphilis

**Published:** 1896-04

**Authors:** E. Van Goidtsnoven

**Affiliations:** Atlanta, Ga.


### NOTE ON THE HISTORY OF SYPHILIS.

BY E. VAN GOIDTSNOVEN, M.D.,

ATLANTA, GA.

Syphilis is composed of two words—*sys*, a sow, and *phileo*, I love. This appellation was coined by Fracastor, a celebrated physician of Verona. He wrote a Latin poem on this terrible disease in the year 1530. Previous to that time syphilis, in common with many other diseases, had no scientific name, but was designated in various countries according to their respective languages and dialects expressive of the disease, the effects it produced, and the terror it inspired. In France it was called *paillardise*.

Some writers, amongst them Bell and Cazenave, contend it raged severely amongst the ancients. Some describe it as a form and degeneration of leprosy, whilst others—and these constitute the great majority—assert that it is of modern origin, for according to their statement, its first appearance in Europe occurred in 1494. Those who have adopted the latter opinion point out Naples as the cradle of syphilis during the French occupation of that city. Oviedo and his disciples assert that this disease was imported from the New World by the soldiery of Christopher Columbus. Astruc, a writer of considerable repute on venereal diseases, was a strong advocate of this theory. He also attempted to demonstrate with remarkable erudition that syphilis was entirely unknown to the physicians and historians who wrote before the fifteenth century, and that it was not and could not be a degenerative process from leprosy. This opinion was subsequently combated by Sanchez in a masterly manner. Cazenave, a high authority on syphilis, compiled a long series of documents from the Leviticus of Moses up to the authors of the fifteenth century, giving numberless descriptions which seem to characterize some of the accidents of syphilitic origin. Littré, in his *Gazette Medicale,* proves that syphilis existed in France in the thirteenth century and presented forms analogous to those we meet with to-day.

Police regulations have been found in the archives of London bearing the date of 1162, having for their object the prevention of syphilitic propagation. Similar regulations were found in Venice dated 1302. The most remarkable document for the prevention of syphilis was issued in 1347 in the city of Avignon by Joan Queen of Sicily and Countess of Provence. It reads as follows: The Queen orders that on every Saturday a matron and a barber appointed by the consuls visit all the debauched women of the bagnios, and if any one of these be found afflicted with the avenging disease of paillardise, the same shall be removed and assigned some special and private lodging, there to be unknown and inaccessible to any one, so that the youth be spared any evil."

This is a remarkable document. It disproves the Neapolitan origin of syphilis beyond all doubts. On the other hand, it obviously shows that medical science, lore, practice, and ethics were in a state verging on degradation no further back than the fourteenth century. Think of a royal decree appointing a barber and an ignorant woman, in the very heart of christendom, to diagnose a case of one of the vilest and most intricate diseases that has ever afflicted humanity!

No wonder we have such poor materials and meagre data to rely upon!

The existence of a disease antedates its recognition, even under the glare of modern science! *Vide* appendicitis!

---

YOUNG PHYSICIAN (wishing to feel his fair patient's pulse)—Will you give me your hand?

Fair patient (embarrassed)—Oh, but, doctor, you--you know—I am so unprepared! I must really ask papa first.

